# Comparison of various continence definitions in a large group of patients undergoing radical prostatectomy: a multicentre, prospective study

**DOI:** 10.1186/s12894-019-0500-6

**Published:** 2019-07-25

**Authors:** Sigrun Holze, Meinhard Mende, Karl V. Healy, Norbert Koehler, Lutz Gansera, Michael C. Truss, Udo Rebmann, Stephan Degener, Jens-Uwe Stolzenburg

**Affiliations:** 1 Department of Urology, University of Leipzig, Liebigstr. 20, 04103 Leipzig, Germany; 2 Coordination Centre for Clinical Trials, University of Leipzig, Härtelstr. 16-18, 04107 Leipzig, Germany; 3 Department of Medical Psychology and Medical Sociology, University of Leipzig, Philipp-Rosenthal-Str. 55, 04103 Leipzig, Germany; 4 Department of Urology, Klinikum Dortmund, Beurhausstr. 40, 44137 Dortmund, Germany; 5 Department of Urology, Diakonissenkrankenhaus Dessau, Gropiusallee 3, 06846 Dessau, Germany; 6 Department of Urology, Helios Klinikum Wuppertal, Heusnerstr. 40, 42283 Wuppertal, Germany

## Abstract

**Background:**

Due to the usage of various measurement methods and definitions, comparing continence rates after radical prostatectomy is a challenging task. This study compares continence rates based on different methods and aims to identify the definition for continence which agrees best with the patients’ subjective assessment of continence.

Additionally, continence was controlled for multiple influencing factors.

**Methods:**

This prospective multicentre study was carried out in seven hospitals throughout Germany. Before and at 3, 6, and 12 months after surgery self-reporting questionnaires were completed and returned by 329 (84.4%) of 390 eligible patients. The questionnaires were independently evaluated and analysed by a third party. Association of continence with demographic, operative, and tumour factors in an ongoing comprehensive prostate cancer database was evaluated.

**Results:**

The continence rate drops substantially for patients undergoing radical prostatectomy but increases again with time. Concrete numbers vary considerably depending on definition – 44% at 3 months and 68% at 12 months after surgery (0 pads) vs. 71 and 90% (0–1 pads). Significant confounding variables regarding continence rate are nerve-sparing procedure, categorized Gleason score, rehabilitative cure treatment, and pelvic floor training.

The definition of 0 pads for continence coincides greater than 0–1 pads with the patients’ self-assessment of being continent.

**Conclusion:**

A standardized definition for continence would be desirable, as it is one of the most important preconditions to guarantee sound comparison of continence rates. Since there are enough other factors that make comparison difficult, we suggest using the definition of “0 pads”. It is easily measured objectively, leaves no room for interpretation, and agrees best with the patients’ self-assessment.

**Electronic supplementary material:**

The online version of this article (10.1186/s12894-019-0500-6) contains supplementary material, which is available to authorized users.

## Background

In Northern and Western Europe the carcinoma of the prostate is the third most frequent type of cancer and by far the most prevalent cancer among men. 128 and 140 out of 100,000 men are diagnosed with this disease, respectively [1]. As a curative treatment, radical prostatectomy (RP) is widely used.

Although RP assures a low morbidity rate [2], is very effective in reducing mortality [3], and the amount of postoperative complications have been reduced due to advances in surgical technique [4], treatment modalities generally cause two major side effects: urinary incontinence and erectile dysfunction [5–7]. Both have a considerable negative impact on the patients’ postoperative quality of life [8, 9]. The former even more so than the latter [10]. Consequently, there is a large interest in evaluating incontinence’s postoperative extent and its influencing factors (e.g., [11–14]).

However, no generally shared standard definition of continence exists. This strongly affects the reported number of patients considered continent. One meta-analysis has found continence rates ranging as far as 67 percentage points (from 5 to 72%) which has been attributed mainly to varying definitions [15]. This inconsistency in defining continence, exacerbated by the heterogeneity of used study designs and samples, data collection methods, measuring instruments, and lengths of follow-up, makes it extremely difficult to compare published results. This has been widely recognized among contemporary research literature (e.g., [16–18]). This paper’s main objective is then to identify the best possible definition of continence and thus help prevent the distortion of reported outcomes. Secondary objectives are to investigate the development of the post-surgical continence rate and reveal additional factors that influence continence after RP.

Written informed consent was obtained from all patients prior to their participation. The study was approved by the Ethics Committee of the University of Leipzig, Faculty of Medicine (approval no. 219–2007) and has therefore been performed in accordance with ethical standards.

## Methods

### Study design

In this prospective longitudinal multicentre study patients with newly diagnosed localised prostate cancer undergoing radical prostatectomy were recruited between February 2008 and May 2009 from seven hospitals in Germany. Patients with other cancer types, pathological cancer stage of pT4, dementia, psychosis, or insufficient knowledge of the German language were excluded. Patients were initially asked to fill out a questionnaire in the hospital one day before surgery in hospital (baseline). Subsequent questionnaires were sent three, six, and twelve months after surgery via mail. These follow-up questionnaires are different from those necessary to fulfil the requirements of certified prostate cancer centres.

There have already been articles published based on the data of this study [19, 20] which have found no significant distinction in comparing the urinary continence rates of endoscopic extraperitoneal radical prostatectomy (EERPE) with open radical prostatectomy (ORP). Our analysis of the influence of continence definition on continence rates will add to these findings.

Continence data were evaluated as followed: (a) number of pads used per day (b) patients’ subjective assessment of continence and (c) urinary symptom score according to the quality of life questionnaire EORTC QLQ-PR25.

Only patients reporting full continence prior to surgery were included in the analysis. Socio-demographic data, clinical data, prostate cancer characteristics (e.g. clinical stage, prostate-specific antigen, biopsy Gleason score, positive surgical margins), and surgical features (surgical approach, nerve-sparing) were recorded. Socio-demographic data were retrieved from the questionnaire and clinical data were compiled from the respective patient’s record.

### Patients

In total, 487 patients treated with RP were eligible to participate in the study. Ninety-seven patients refused study participation at baseline. Thus, the total number of valid preoperative questionnaires was 390 (baseline participation rate: 80.1%). Three hundred twenty-nine patients completed and returned a second, third, and fourth questionnaire at 3, 6, and 12 months after surgery (participation rate: 84.4%). Sixty-one patients did not fill out the questionnaires.

There were no statistically significant differences between respondents (study participants) and non-respondents (patients who were excluded from or refused participation) regarding age (65.3 vs. 64.8 years) and pelvic lymph node dissection (75% vs. 76%). The number of patients who underwent nerve-sparing surgery was significantly higher among respondents than non-respondents (64% vs. 55%, *p* < 0.05).

### Instruments

For the assessment of urinary incontinence after radical prostatectomy, different criteria were used:

- Number of pads used in a 24-h period: Patients were categorized as either (completely) continent (requiring 0 pads), socially continent (0–1 pad per day), or incontinent (2 or more pads per day).
- Patient’s self-assessment of continence: Patients answered the question: Do you suffer from urinary incontinence? (yes/no).
- Urinary and bowel symptoms: These are two scales from the validated EORTC QLQ-PR25 – a multidimensional questionnaire of the “European Organization for Research and Treatment of Cancer” to measure the prostate related quality of life [21]. The QLQ-PR25 was scored according to the EORTC scoring manual [22]. A high score on these 0–100 point scales indicates a high burden of symptoms.

### Statistical methods

Continence was defined as 0 pads per day. Agreement between the different criteria for continence (“0 pads” vs. “0–1 pad”) and the subjective assessment via questionnaire was measured by Cohen’s Kappa. Kappa values between 0.61 and 0.8 indicate good agreement [23, 24].

The trial cohort was characterized by mean ± standard deviation for continuous and frequencies / percentages [Wilson 95% C.I.] for categorical data. Means of continuous data were compared by t-test (Welch) for independent samples, frequencies by chi-square or Fisher’s exact tests, if appropriate.

With regard to continence in the follow-up, we juxtaposed the three categories 0, 1 and 2 and more pads at 3, 6 and 12 months. These frequencies were compared by McNemar test. We modelled continence at 3 months on the one hand by a simple logistic regression and on the other hand by a multiple model with all detected confounders (cf. Table 1).

**Table 1:** Sociodemographic and clinical characteristics of the study population

Sociodemographic	Total	Mean /	
	*N* = 329	Proportion	95% CI
Age (Mean, SD)	329	65.3	[65, 65.7]
> 65 years	180	54.7%	[49.3, 60%]
Family Status: Married	283	86.0%	[81.9, 89.4%]
Partnership: Yes	314	95.7%	[92.6, 97.2%]
School Education: Higher Level	135	41.4%	[35.9, 46.4%]
University Education	124	38.2%	[32.6, 43%]
Health Insurance: Private	66	20.4%	[16.1, 24.7%]
Employment Status			
Employed	92	29.0%	[23.4, 33%]
Pensioner	225	71.0%	[63.2, 73.2%]
Clinical			
PSA ng/ml (Mean, SD)	329	8.4	[8.1, 8.8]
Surgery	152	46.2%	[40.9, 51.6%]
EERPE	177	53.8%	[48.4, 59.1%]
ORP			
Nerve-sparing			
none	119	36.2%	[31.2, 41.5%]
unilateral	57	17.3%	[13.6, 21.8%]
bilateral	153	46.5%	[41.2, 51.9%]
Pelvic Lymph Adenectomy	243	74.8%	[68.9, 78.3%]
Pathological Stage			
pT1a - pT1c	4	1.2%	[0.5, 3.1%]
pT2a - pT2c	235	71.4%	[66.3, 76%]
pT3a - pT3b	90	27.4%	[22.8, 32.4%]
Positive Surgical Margins			
R0	278	84.8%	[80.2, 88%]
R1	50	15.2%	[11.7, 19.5%]
Gleason Score			
Gleason ≤6	131	40.1%	[34.7, 45.2%]
Gleason 7	158	48.3%	[42.7, 53.4%]
Gleason 8–10	38	11.6%	[8.5, 15.5%]

The search for covariates multiple associated with continence at 3 months was done in several steps. We started with variables potentially associated with continence: therapy (ORP vs. EERPE), nerve-sparing technique, PSA, Gleason score (categorized max. 6 / 7 / 8 and more), age, TNM stage, pelvic lymph node dissection, pelvic floor training at 3 months, additional therapy, medical rehabilitation and school and university education (cf. Table 1). A LASSO procedure [25] was applied to select variables for fitting a multiple logistic model. While setting λ2 = 0, the optimal λ1 was found by maximising the cross-validated likelihood. We excluded further variables only weakly associated with continence to get a “sparse” and well interpretable standard logistic regression model for estimating and testing. The results of this model were depicted by means of a Forrest plot.

We performed data preparation and basis statistics by IBM SPSS Statistics, version 22. The LASSO procedure and the generation of the Forrest plot was done by R (R Core Team, Vienna, Austria) [26]. Alpha = 5% was globally determined as two-sided significance limit.

## Results

### Socio-demographic and clinical factors

Table 1 characterises our study population.

The mean (SD, range) age of the patients was 65.3 (6.4, 45–81) years. One hundred forty-nine patients (45.3%) were ≤ 65 years and 180 patients (54.7%) were >  65 years (cf. Table 1). Two different surgery methods were used: EERPE and ORP. One hundred and fifty-two patients (46.2%) were operated on using the former procedure, 177 (53.8%) using the latter. In total, 63.8% of patients received a nerve-sparing procedure. Of these, 72.9% received a bilateral nerve-sparing prostatectomy. Two hundred and eighty-three patients (86%) were married. One hundred thirty-five patients (41.4%) had an education of a higher level.

### Continence rates at 3, 6, and 12 months

**Fig. 1:**
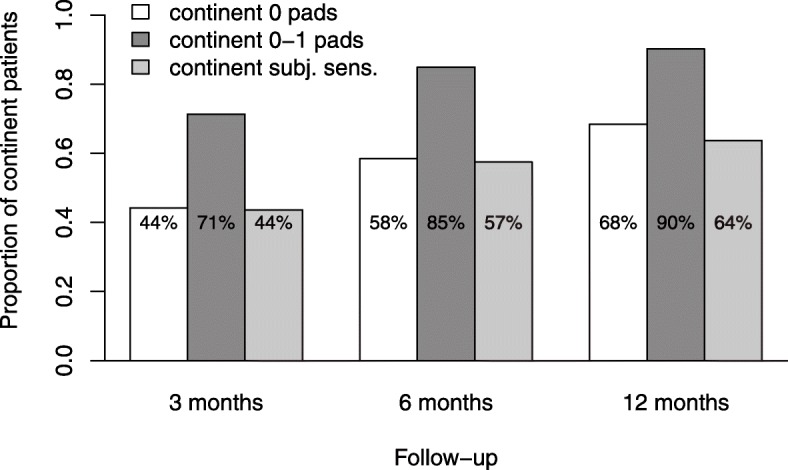
Different assessments of continence

The analysis shows that the continence rate (0 pads) at 3 months after surgery increases significantly from 44% to 6 months (59%, *p* < 0.001) and 12 months (68%, *p* < 0.001, see Fig. 1, Additional file 2: Table S2). The continence rates were significantly higher (*P* = 0.001) after nerve-sparing surgery (3 months: 51%, 6 months: 68%, 12 months: 78%) compared to non-nerve-sparing surgery (31, 42, 52%) (not in Figure).

The social continence rate (being defined as 0–1 pad) at 3, 6, and 12 months was significantly higher (71, 85, 90%, *P* < .05) than the rates of complete continence (0 pads) and the continence rate according to the patients’ self-assessment (44, 57, 64%, cf. Fig. 1).

### The best definition of continence: comparison of different criteria

The high diversity of applied continence measures complicates the comparison of results. This is why this study aims to identify criteria of continence which match best the patients’ perception of continence. This was accomplished by collecting the number of pads as well as the patients’ subjective assessment of continence.

It is controversially discussed if continence should be defined as the usage of “0 pads” or “0–1 pad”. To clarify this issue, we calculated kappa coefficients measuring agreement between subjective sense of continence with assessment by the 0 and 0–1 criterion (cf. Table 2).

**Table 2:** Agreement between the different criteria and the subjective sense of continence at 3 (6 and 12) months

0/1-pads criterion					
Subjective sense of continence	continent 0–1 pads	incontinent 2+ pads	total	follow-up	kappa (95% c. i.)
Continent	140	3	143	3 mo.	0.44 (0.36, 0.52)
Incontinent	94	91	185	6 mo.	0.31 (0.22, 0.40)
total	234	94	328	12 mo.	0.31 (0.22, 0.40)
0-pads criterion					
Subjective sense of continence	continent 0 pads	incontinent 1+ pades	total	follow-up	kappa (95% c. i.)
Continent	122	21	143	3 mo.	0.73 (0.65, 0.80)
Incontinent	23	162	185	6 mo.	0.70 (0.62, 0.78)
Total	145	183	328	12 mo.	0.64 (0.56, 0.73)

Left panel: Agreement of subjective sense of continence (in rows) with actual use of pads at 3 months, above: 0/1-criterion, below: 0-pads criterion

Right panel: Estimated measure of agreement (kappa coefficient incl. 95% confidence interval) between subjective sense of continence and actual use of pads after 3, 6 and 12 months

Kappa coefficients for 0 pads are higher than 0.61, indicating good agreement. At 3 and 6 months, even the lower 95% confidence bounds are over this threshold. On the other hand, kappa = 0.44 and 0.31 show only moderate to weak agreement of the 0–1 pad criterion with the subjective assessment of continence.

Table 2 (left panel) comparing the subjective assessment (in rows) with the evaluation by the respective criterion at 3 months (in columns) may illustrate this. The counts in the main diagonal are the numbers of patients for whom the subjective and objective assessments agree. That is, 140 and 122 patients feel continent and the 0–1 and 0 pads criterions respectively assess them continent, too. In the same manner, 91 and 162 patients feel incontinent in agreement with the evaluation by the 0–1 and 0 pads criterions. However, there are 94 patients feeling incontinent even though the 0–1-pads criterion determines them as continent. To summarize, there are 94 + 3 = 97 / 326 (30%) discrepant estimates by the 0–1 criterion in comparison to only 21 + 23 = 44 / 326 (13%) discrepant estimates by the zero pad criterion. The results at 6 and 12 months are similar.

Even though the 0 pads criterion is not perfect, it reflects the subjective sense of continence much better than the criterion of a safety pad (0–1 pads).

### Factors associated with patients’ post-surgical continence status

**Fig. 2:**
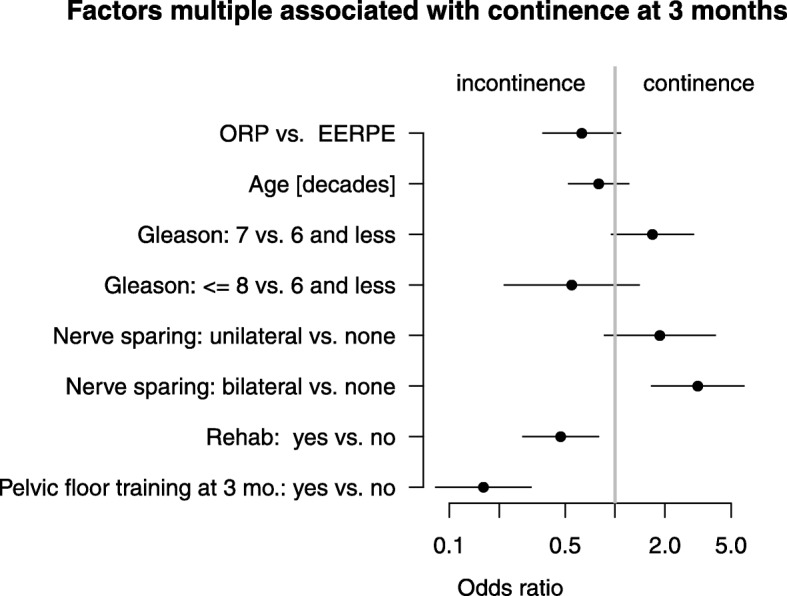
Multivariate associations of several factors with continence at 3 months

In our multivariate analyses, we observed a small disadvantage, albeit non-significant, of the ORP procedure compared to EERPE with respect to the binary endpoint continence at 3 months (odds ratio (OR) = 0.71, 95%C.I. 0.45–1.10). This alleged small disadvantage of ORP is only marginally changed by adjustment for confounders (OR = 0.63 [0.37–1.08], *p* = 0.092). TNM stage and PSA pre surgery, additional therapy, pelvic lymph node dissection, as well as social factors (marital status, in partnership or not, school and university education and employment state) are not predictive for continence. Thus, we got our final model with six variables after removing these confounders, but leaving age (cf. Fig. 2).

A second important question is which variables are bivariate and multiple associated with continence.

Of all covariates, the clear advantage of the nerve-sparing procedure is obvious. However, only the advantage of bilateral versus non-nerve-sparing technique is significant (OR 3.15 [1.55–5.98], *p* < 0.001). We could also observe a positive effect of the unilateral nerve-sparing procedure on the continence recovery rate (OR 1.86 [0.86–4.02] vs. non nerve-sparing, *p* = 0.113). However, there is no statistical evidence for this observation. Categorized Gleason score is significantly associated (*p* = 0.029), but none of the categories (Gleason =7: OR 1.68 [0.95–2.97], *p* = 0.073 against the reference category Gleason at most 6, Gleason ≥8: OR 0.55 [0.22–1.40], *p* = 0.208). Higher age is very weakly associated with risk of incontinence (OR 0.80 [0.53–1.21], *p* = 0.285). That is, odds for continence decrease by a factor of 0.8 per 10 years.

Finally, rehabilitative cure treatment (OR 0.47 [0.28–0.80], *p* = 0.005) and pelvic floor training (OR 0.16 [0.08–0.31], *p* < 0.001) are significantly negatively correlated with continence. Interpretation is difficult because statistics do not discriminate between risk factors for and responses on an outcome. We assumed that patients who are incontinent at 3 months are encouraged to participate in pelvic floor training. To check this assumption, we contrasted the continence rates at 6 and 12 months of patients who participated in pelvic floor exercises at three months vs. those who did not participate. Our data clearly shows the considerably steeper increase of continence rate at 6 and 12 months of patients who formerly exercised their pelvic floor (Additional file 1: Figure S1). This supports our hypothesis. The relationship of continence and rehabilitation could be similar. Only half of the patients continent at 3 months participated in rehabilitation (51%) but nearly ¾ (74%) of the incontinent patients. In other words, the portion of incontinence at three months is 65% in patients who participated in rehabilitation and only 40% in those who did not (OR 2.8). This difference in incontinence rates decreases up to six months (47% vs. 31%), giving an odds ratio of 1.9. This OR does not change essentially up to 12 months (37% vs. 22%, OR 2.1). This indicates that a fraction of patients, irrespective the method of surgery, has bad conditions for continence in spite of rehabilitation.

### Urinary and bowel symptoms: EORTC QLQ-PR 25

There was a significant and clinically relevant worsening in urinary symptoms from a baseline score of 21.7 to 31.6 (− 9.9) points at 3 months (*P* < .001, Additional file 1: Table S1). Based on previous QoL research, a difference of at least 10 mean score points between different points in time is considered clinically relevant [27–29]. There was a gradual reduction of symptoms at 6 and 12 months after surgery.

In contrast, the burden of bowel symptoms was generally very low at all time-points. There was no statistically significant change over time.

## Discussion

There are different reasons for the discrepancy between reported continence rates. Different clinical and sociodemographic characteristics of patients or the experience of the surgeon may be influencing factors [13, 30–32]. However, according to Sacco et al. [33] and Borregales et al. [16], it is the heterogeneity of methods and definitions that have the largest impact on the results. Trials using patient questionnaires for the evaluation of postoperative incontinence report lower continence rates than trials based on the physicians’ assessment. For example, Lee et al. [34] have found continence rates varying from 14.7%, based on reports by patients, to 51.5%, based on reports by physicians at the same time. These numbers emphasize the problem of comparing continence rates if different measuring methods and/or definitions are used.

Some publications include patients using 1 pad per day in the group of complete continence. In this study, the strict definition of complete continence (0 pads) was used. Conflictingly, Krupski et al. [35] argue for a composite score to define continence. They find the 0 pads definition to be too superficial. An in-depth evaluation could surely reflect better the complex issue that is continence, but at the same time it would complicate the comparison of different study results. Krupski et al. acknowledge this themselves. Therefore, we strongly suggest the simple, yet conclusive definition of 0 pads per day. But keeping this objection in mind, the patients’ self-assessment can be regarded as valuable supplemental information. Additionally, the standardized questionnaire EORTC QLQ-PR25 has been used as a part of this study to collect information on different aspects of urinary incontinence and its impact on the patients’ quality of life for a deeper understanding. Nonetheless, other authors agree with adopting 0 pads per day as a standard. Borregales et al. [16] have systematically reviewed several articles on the subject and believe this definition to be optimal. Liss et al. [36] come to the conclusion that since there is a significant increase in quality of life with 0 pads in opposition to usage of 0 to 1 or a safety pad, this definition should be assumed universally. Incontinence and urinary symptoms are frequently associated with restrictions of social contacts and activities and have a high influence on the patients’ quality of life. However, as our data also show, incontinence after RP is often a temporary problem. In the majority of patients, these symptoms decrease or disappear during the first postoperative year [37].

Patients who underwent nerve-sparing surgery were significantly less affected by urinary symptoms than patients who underwent non-nerve-sparing surgery. This effect can be observed for both unilateral and bilateral nerve-sparing techniques but is only of statistical significance for the latter. In regards to the effect of pelvic floor training, some articles state a positive outcome on the continence rate [38, 39]. A review conducted by Hunter et al. [40], on the other hand, elicits conflicting results. One of the seven articles considered in the review regarding this issue is in concordance with the aforementioned articles, “whereas the estimates from the others were consistent with no effect” [40] of pelvic floor training on continence rate. In our differentiated analyses, a positive correlation can be observed beyond the point of 3 months post-surgery. To assume that the negative correlation before that point in time is an artefact which results from increased training when incontinence stays consistent seems reasonable. Since the effect of rehabilitative cure treatment is most likely a mediator of pelvic floor training, similar assumptions can be made concerning its negative correlation with continence.

These results clearly show the superiority of the 0 pads usage definition. Firstly, defining continence as the usage of 0 pads has a higher objectivity and secondly, it shows good agreement with subjective assessments of continence.

## Conclusion

Looking for a uniform continence criterion, the 0-pads criterion is advisable. It is clear and objective and does reflect the subjective sense of continence better than the criterion of 0–1 pads. We found the bilateral nerve-sparing procedure as only covariate clearly associated with continence.

## Additional files

Additional file 1: **Figure S1.** Continence rates for patients who executed pelvic floor training or not at 3 months. **Table S1.** Symptom scales of the EORTC QLQ-PR25. (DOCX 70 kb)

Additional file 2: **Table S2.** Number of pads in the follow-up. (DOCX 15 kb)

## Data Availability

Unfortunately, the datasets used for this article cannot be made publicly available at this point in time due to the lack of an adequate publishing platform. The data can be received from the corresponding author or the biometrician upon request.

## Abbreviations

EERPE: Endoscopic extraperitoneal radical prostatectomy
EORTC QLQ: European Organization for Research and Treatment of Cancer Quality of Life Questionnaire
OR: Odds ratio
ORP: Open radical prostatectomy
RP: Radical prostatectomy
