# Supplementary material for: Comparison of various continence definitions in a large group of patients undergoing radical prostatectomy: a multicentre, prospective study
Source: BMC Urol. 2019 Jul 25;19:70. doi: 10.1186/s12894-019-0500-6 (PMC6659208; doi:10.1186/s12894-019-0500-6)
Supplement: Supplementary file 1 — Figure S1. Continence rates for patients who executed pelvic floor training or not at 3 months. Table S1. Symptom scales of the EORTC QLQ-PR25. (DOCX 70 kb) [file 12894_2019_500_MOESM1_ESM.docx]

**Additional file 1**

Figure S1- supplementary: Continence rates for patients who executed pelvic floor training or not at 3 months

**Table S1: Symptom scales of the EORTC QLQ-PR25**

| **EORTC-Scale** | **baseline** | **3 months** | **6 months** | **12 months** |
| --- | --- | --- | --- | --- |
|  |  |  |  |  |
| **Urinary Symptom scale** | 21.7 ± 16.6 | 31.6 ± 19.7 | 26.8 ± 19.0 | 24.1 ± 18.5 |
| Change | - | -9.9^1)^ | +4.8 | +2.1 |
| P Value | - | <0.001 | <0.001 | <0.001 |
| **Bowel Symptom scale** | 3.9 ± 8.4 | 4.8 ± 9.1 | 5.0 ± 10.4 | 5.7 ± 10.3 |
| Change | - | -0.9 | -0.2 | -0.7 |
| P Value | - | 0.121 | 0.597 | 0.234 |

N = 329, Mean scale scores ± standard deviation; t-test (2-tailed).

Score range from 0 to 100. A higher score for the Symptom scales represents more severe symptoms. Changes were calculated as the scale score at earlier timepoint minus scale score at later timepoint (e.g. ^1)^ scale score at baseline minus scale score at 3 month postoperatively). Negative (-) changes means diminished QOL/more symptoms. Positive (+) changes in any scale means improved QOL/less symptoms.
