# Supplementary material for: Comparison of various continence definitions in a large group of patients undergoing radical prostatectomy: a multicentre, prospective study
Source: BMC Urol. 2019 Jul 25;19:70. doi: 10.1186/s12894-019-0500-6 (PMC6659208; doi:10.1186/s12894-019-0500-6)
Supplement: Supplementary file 2 — Table S2. Number of pads in the follow-up. (DOCX 15 kb) [file 12894_2019_500_MOESM2_ESM.docx]

|  |  | EERPE vs. RRPE | | | | | | |
| --- | --- | --- | --- | --- | --- | --- | --- | --- |
| Visit |  | EERPE | | RRPR | | Total | | |
| 3 months | 0 pads | 74 | 48.7% | 71 | 40.3% | 145 | 44.2% |  |
|  | 1 pad | 40 | 26.3% | 49 | 27.8% | 89 | 27.1% |  |
|  | 2+ pads | 38 | 25.0% | 56 | 31.8% | 94 | 28.7% |  |
|  | Total | 152 | 100.0% | 176 | 100.0% | 328 | 100.0% |  |
| 6 months | 0 pads | 99 | 65.6% | 91 | 52.3% | 190 | 58.5% |  |
|  | 1 pad | 37 | 24.5% | 49 | 28.2% | 86 | 26.5% |  |
|  | 2+ pads | 15 | 9.9% | 34 | 19.5% | 49 | 15.1% |  |
|  | Total | 151 | 100.0% | 174 | 100.0% | 325 | 100.0% |  |
| 12 months | 0 pads | 112 | 74.2% | 111 | 63.4% | 223 | 68.4% |  |
|  | 1 pad | 29 | 19.2% | 42 | 24.0% | 71 | 21.8% |  |
|  | 2+ pads | 10 | 6.6% | 22 | 12.6% | 32 | 9.8% |  |
|  | Total | 151 | 100.0% | 175 | 100.0% | 326 | 100.0% |  |

Table S2: Number of pads in the follow-up
